# A methodology for assessing the effect of localizer orientation on the consistency of tube current modulation in CT

**DOI:** 10.1093/rpd/ncaf190

**Published:** 2026-03-13

**Authors:** Daniel Thor, Marcus Söderberg, Gavin Poludniowski, Torkel B Brismar

**Affiliations:** Department of Nuclear Medicine and Medical Physics, Karolinska University Hospital, SE-141 83 Stockholm, Sweden; Department of Oncology-Pathology, Karolinska Institutet, SE-141 83 Stockholm, Sweden; Department of Translational Medicine, Medical Radiation Physics, Skåne University Hospital, Lund University, SE-205 02 Malmö, Sweden; Department of Hematology, Oncology and Radiation Physics, Radiation Physics, Skåne University Hospital, SE-205 02 Malmö, Sweden; Department of Nuclear Medicine and Medical Physics, Karolinska University Hospital, SE-141 83 Stockholm, Sweden; Department of Clinical Science, Intervention and Technology, Karolinska Institutet, SE-141 83 Stockholm, Sweden; Department of Clinical Science, Intervention and Technology, Karolinska Institutet, SE-141 83 Stockholm, Sweden; Department of Radiology, Karolinska University Hospital in Huddinge, SE-141 83 Stockholm, Sweden

## Abstract

Localizer orientation can significantly impact the automatic tube-current modulation (ATCM) in computed tomography (CT). The purpose of this study was to introduce a methodology for assessing ATCM consistency and to compare the outcomes in image noise and radiation dose between a single anterior–posterior (AP) localizer and a combination of lateral and AP (LAT + AP) localizers. A total of 299 prospective patients referred for a routine CT thorax examination were randomly assigned to a single AP or LAT + AP localizer. Measurements of image noise and patient size were performed in the thorax and liver, and the corresponding effective milliampere-second (mAs_eff_) at these positions were collected. Performance analysis was conducted using the normalized root mean squared error (nRMSE) of mAs_eff_ and image noise relative to patient size. A smaller nRMSE indicates more consistent inter-patient variance in image noise and mAs_eff_ for a given size. The LAT + AP group had a statistically significant lower nRMSE for mAs_eff_ and image noise in the thorax region, and for mAs_eff_ (but not image noise) in the liver region. A post-hoc finding indicated that a subgroup of female patients with laterally protruding breasts was overexposed (+57% radiation dose) in the thorax region when using the AP localizer. Using LAT + AP localizer resulted in more consistent image noise levels and radiation doses compared to single AP localizer. The effect was small, except for the subgroup of females, where radiation doses were higher in the AP group. The findings are specific to the CT system used in this study and may not be generalizable to other CT models or software versions. Nonetheless, the proposed methodology provides a valuable approach for evaluating ATCM consistency.

## Introduction

Today, most computed tomography (CT) systems are equipped with automatic tube current modulation (ATCM) to adjust X-ray exposure based on patient size, thereby reducing radiation dose while maintaining consistent intra- and inter-patient image quality [1]. ATCM typically utilizes a so-called localizer radiograph to estimate patient size and calculate the appropriate exposure.

The default setting for body scan protocols is often a single anterior–posterior (AP) localizer or, in some cases, a posterior–anterior (PA) localizer. Some institutions also acquire an additional lateral (LAT) localizer. The choice of localizer orientation systematically biases the estimation of patient size, leading to variations in average dose levels for a given reference exposure (e.g. quality reference mAs or image noise index) [2–4]. This bias can be offset by adjusting the reference level to maintain a consistent average value, either through phantom studies or by comparing patient dose averages. However, even when the averages are equal, the variance across different orientations may differ. One orientation could potentially yield more consistent results, i.e. lower inter-patient variance in image noise and effective milliampere-second (mAs_eff_) adjustment as a function of patient size. Consistency is advantageous in image optimisation, as lower variance in image noise facilitates the identification of specific trends (e.g. poor image quality in larger patients).

The purpose of this study was to introduce a methodology for assessing ATCM consistency and to compare the outcomes in image noise and radiation dose (mAs_eff_) between a single AP localizer and a combination of LAT and AP localizers.

## Materials and methods

### CT

Two identical Siemens CT scanners (Somatom Definition Flash, Siemens Healthineers, Forchheim, Germany) were used. These systems are equipped with longitudinal and angular automatic tube current modulation (ATCM) software, known as CARE Dose 4D (software version VA44A). The software utilizes the localizer to prescribe longitudinal tube current modulation, in combination with real-time feedback from the detector during the scan to adjust modulation in the angular direction. The attenuation profile along the patient’s longitudinal axis is derived from the localizer and mathematically transformed to estimate the corresponding profile in the perpendicular projection. Angular modulation is based on attenuation profiles acquired during each tube rotation, with the tube current for the subsequent rotation adjusted after a half-rotation delay. When two localizers of different orientations are used, information from both images is incorporated into the modulation process [4, 5].

By using the adaptation strength settings in CARE Dose 4D, the user can control how strongly the system modulates the tube current in relation to patient size and attenuation. CARE Dose 4D was configured with adaptation strength settings of average for slim patients and strong for obese patients (average/strong). These settings have a more similar mAs-adjustment slope for slim and large patients as compared to settings average/average and strong/strong (according to the technical reference manual [6]. This configuration was chosen to make it more suitable for the intended analysis.

### Study approach

Patients examined with the routine thorax protocol (Table 1) of the department were prospectively included in the study. One group was scanned using a single AP localizer, in line with both the department’s standard practice and the manufacturer’s default setting, while a second group was scanned using both LAT and AP localizers. The analysis of the ATCM performance for the AP- versus LAT + AP-group was done by fitting curves of mAs_eff_ and image noise against patient size. The normalized root mean squared error (nRMSE) of the residuals was used as the main outcome metric. A higher nRMSE indicates a higher variability and less consistent inter-patient mAs_eff_ and image noise in clinical practice.

**Table 1:** Overview of scanning parameters.

**Localizer settings:**	
Tube voltage (kV)	120
Tube current (mA)	20
**Spiral settings:**	
Tube voltage (kV)	120
Quality reference mAs (mAs)	AP: 74, LAT + AP: 110
ATCM^a^	CARE Dose 4D
Organ characteristic	Thorax
Detector configuration^b^	128 × 0.6
Detector width (mm)	38.4
Pitch	1.2
Rotation time (s)	0.5
Slice thickness / Increment (mm)	5 / 5
Reconstruction kernel	B31f

^a^Automatic tube-current modulation.

^b^A z-axis flying focal spot technique is used to acquire twice as many projections per rotation as there are detector rows.

To keep average radiation dose levels (CTDI_vol_) similar for the two groups, the quality reference mAs was adjusted in order to cancel out the known effect that localizer orientation can have on CTDI_vol_ [2–4]. For this purpose, a small pilot test was performed on 17 patients to establish such correction factor. In step one, each patient was first planned to be scanned using an AP localizer and the indicated CTDI_vol_ for the thorax scan region was registered. In step two, a LAT localizer was added and the new indicated CTDI_vol_ was registered (note that the order of the localizer does not matter when two localizers are used in CARE Dose 4D). The average change in CTDI_vol_ was −33%, meaning that CTDI_vol_ was lower when both AP + LAT localizers were used compared with the AP localizer alone. Consequently, the quality reference mAs for the single AP localizer group was reduced from 110 to 74. It should be noted that, as of CARE Dose 4D software version VA62/VBxy, this adjustment may no longer be required, as only the AP localizer is directly used for size estimation, with the LAT localizer is used solely to correct for miscentering (if available).

### Data collection

The study was considered a quality assurance study, and ethical approval was therefore not sought prior to initiation. This assessment was subsequently confirmed by the local ethics committee, which waived the application (Stockholm, Sweden; 2017/1848–31/2). All 299 patients examined with the routine thorax CT protocol (as part of one of three examination types: thorax, thorax-abdominal, or thorax-upper-abdominal) during the inclusion periods had their axial 5 mm slices and all localizers automatically transferred to a local dose monitoring DICOM server. All examinations were conducted following the department’s standard positioning protocols, with the radiographer using laser alignment to ensure proper patient centering. Patients were allocated to different groups by systematically adjusting the localizer settings at four time points to minimize the risk of scanner performance drift or other time-related confounders (LAT + AP: May 2016–July 2016, AP: July 2016–March 2017, LAT + AP: March 2017–June 2017, and AP: June 2017–August 2017). No discrimination was made between patients with or without intravenous contrast. Patients with arms positioned down or with metal objects in the field of view (FOV) were excluded. As the distribution of examination types differed between the LAT + AP and AP groups, a rebalancing was performed by randomly excluding patients from the overrepresented groups until the number of examination types was equal in both cohorts, resulting in a total of 299 patients included for analysis.

### Image noise measurements

**Figure 1:**
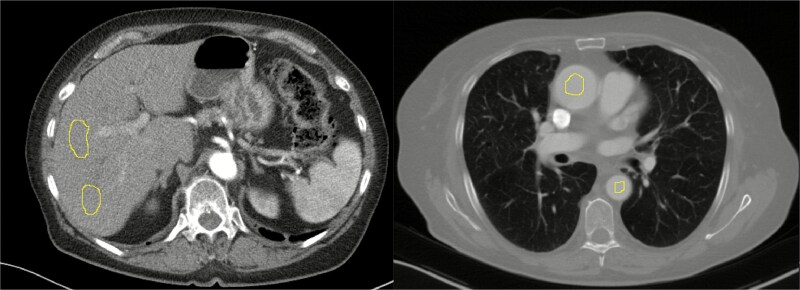
Examples of the two ImageJ free-hand-ROI placements: One in the liver and one within the aorta in the thorax region.

Image noise, defined as the standard deviation of the pixel values, was measured at two positions within the liver parenchyma and two positions within the aorta in the thorax region (Fig. 1). The measurements were carried out manually (by author DT) in ImageJ (Version 1.46, National Institutes of Health, http://imagej.nih.gov/ij). The mean value from two freehand regions of interests (ROI) was used, ensuring that conspicuous vessels in the liver and streak artifacts in the aorta were avoided.

Images from the AP and LAT + AP groups were measured in parallel (alternating) to maintain consistency in manual measurements between groups.

### Patient size measurements

The patients’ water equivalent diameter (D_w_) [7, 8] was calculated from the same image slice as the image noise measurements using a MATLAB script (MathWorks Inc., Natick, MA, USA). The D_w_ script was validated using CT images of CIRS tissue equivalent phantoms of varying sizes (15-y-old, small, medium, and large adult). The calculated D_w_ values were 22.3 cm, 27.2 cm, 30.0 cm, and 36.6 cm, respectively, all within 1% of reported values [8, 9].

Since images were collected from clinical routine, many patients had peripheral areas extending beyond the reconstructed field. In such cases, no D_w_ value was calculated. To account for this, complementary size measurements were performed in the localizer images in the form of localizer profile sums (LPS), defined as the sum of the pixel values across the patient width at the longitudinal position of the image noise measurement (determined via metadata). Before measurement, the localizers were smoothed using a Gaussian filter (MATLAB function: imgaussfilt with sigma = 3), and the mean of 10 pixel rows was used. LPS was measured only in the AP localizer for both groups, even when a LAT localizer was available, to avoid introducing bias. The relationship between LPS and D_w_ was established using linear regression (based on data from both the AP and LAT + AP groups) and subsequently used to impute missing D_w_ values.

### Effective mAs

mAs_eff_ was extracted from the DICOM metadata at the measurement position. It is defined as the product of tube current (mA) and rotation time (s), divided by pitch (mm/mm).

### Constancy control

In addition to the department’s standard quality assurance program, separate tests of image noise levels, CT numbers, and modulation transfer function (MTF) were performed using the manufacturer’s water phantom and built-in evaluation software. The tests were performed at the onset of the study, at each localizer orientation change, and when data collection was finished. The International Electrotechnical Commission (IEC) tolerance levels were applied as pass/fail criteria [10].

### Statistical analysis

Linear regression was performed on the mAs_eff_ and image noise versus patient size using the fitlm function in MATLAB (version R2016a), following data transformation by $\ln \left({mAs}_{eff}\right)$ to achieve a linear relationship. The 95% confidence intervals (CI) of the nRMSE values (i.e. RMSE normalized to the mean value of either the mAs_eff_ or image noise) were estimated using percentile bootstrapping with 3000 iterations and resampling with replacement via the datasample function in MATLAB. To test the null hypothesis (i.e. that the nRMSE of the AP and LAT + AP groups are equal), a distribution of nRMSE differences was generated by computing the difference in nRMSE between the two localizer techniques for each resampled dataset. The 95% CI of the differences in nRMSE was then estimated from this distribution. A statistically significant difference was considered if the 95% CI of the difference did not overlap zero.

## Results

### Patients

Table 2 provides an overview of the measured values for each group. For all metrics related to body size, CT number, and ROI size, the differences in mean values between the groups were ˂6% of the standard deviation.

**Table 2:** Measured values of the primary variables for each group in the liver and thorax regions. Values are presented as mean ± SD, except for number of patients and gender.

	Liver		Thorax	
Group	AP	LAT + AP	AP	LAT + AP
Number of patients	154	145	154	145
Patient gender, males / females	77 / 77	73 / 72	77 / 77	73 / 72
mAs_eff_ (mAs)	86.2 ± 36.6	89.1 ± 33.6	48.7 ± 25.9	52.4 ± 18.9
Image noise (HU)	19.9 ± 3.7	19.1 ± 3.5	16.8 ± 3.5	15.4 ± 2.9
CT-number (HU)^a^	65.4 ± 19.2	67 ± 19.2	267.5 ± 107.2	268.9 ± 103.9
ROI area (mm^2^)^a^	285.7 ± 83.7	285.5 ± 93.9	92.9 ± 31.1	92.5 ± 31.2
D_w_ (cm)^b^	30.7 ± 3.2	30.6 ± 3.2	26.9 ± 2.9	26.9 ± 2.7
AP width (localizer, cm)^a^	n/a	27.8 ± 4.4	n/a	26.1 ± 3
LAT width (localizer, cm)^a^	33.5 ± 3.9	33.5 ± 4.2	36.0 ± 3.6	35.9 ± 4.2
AP width (slice, cm)^a^	25.4 ± 3.9	25.3 ± 3.9	23.5 ± 2.7	23.5 ± 2.6
LAT width (slice, cm)^a^	32.4 ± 3.2	32.4 ± 3.4	34.3 ± 3.4	34.1 ± 3.4

^a^These variables were not part of the analysis but were collected for comparison between groups and are included here for the interested reader.

^b^Including imputed values.

### Patient size measurements

As expected, the correlations between LPS and D_w_ were high (r = 0.95 for the liver region and r = 0.88 for the thorax region). The difference between the estimated D_w_ from the LPS and D_w_ calculated from the axial image was small, with a mean difference of 0.8 cm (2.7%) in the liver region and 1.1 cm (4.2%) in the thorax region. The largest individual differences were 10% and 13%, respectively. The number of imputed D_w_ values were 12 out of 299 in the liver region and 89 out of 299 in the thorax region.

### Main outcome

**Figure 2:**
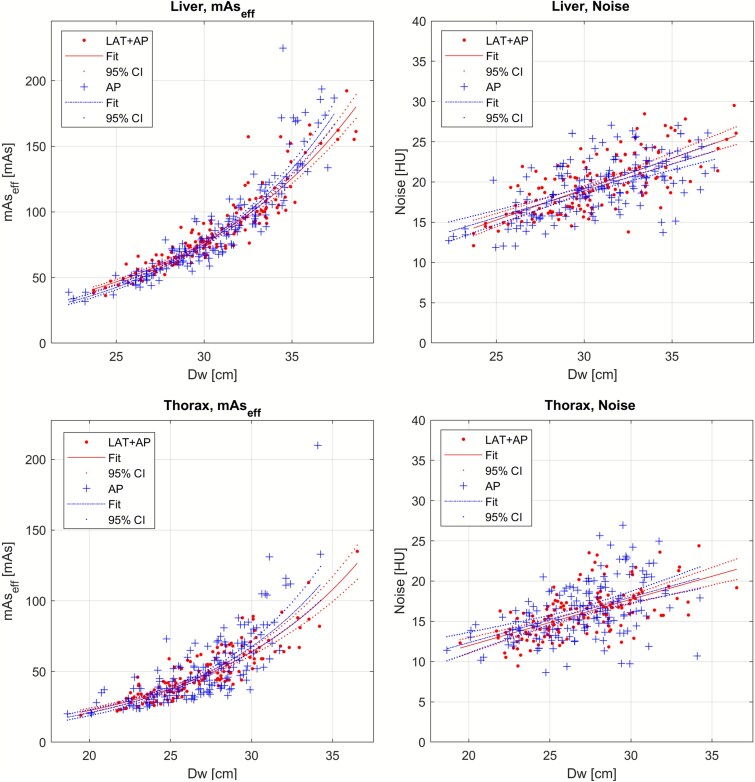
Effective mAs and image noise as a function of D_w_ for both groups in the liver and in aorta within the thorax region. The 95% CI lines correspond to the confidence of the fitted lines and were calculated using MATLAB’s fitlm function. Note: Due to a slight difference in the median mAs_eff_ values between the AP- and LAT + AP-groups, the data points in these graphs have been linearly translated so that the median values for both groups are equal. This adjustment is for visual clarity only (better visual comparison of the scatter plots) and does not affect the analysis. The shifts applied were 3 mAs and 0.6 HU in the thorax data, and 1.8 mAs and 0.5 HU in the liver data, in both directions.

**Figure 3:**
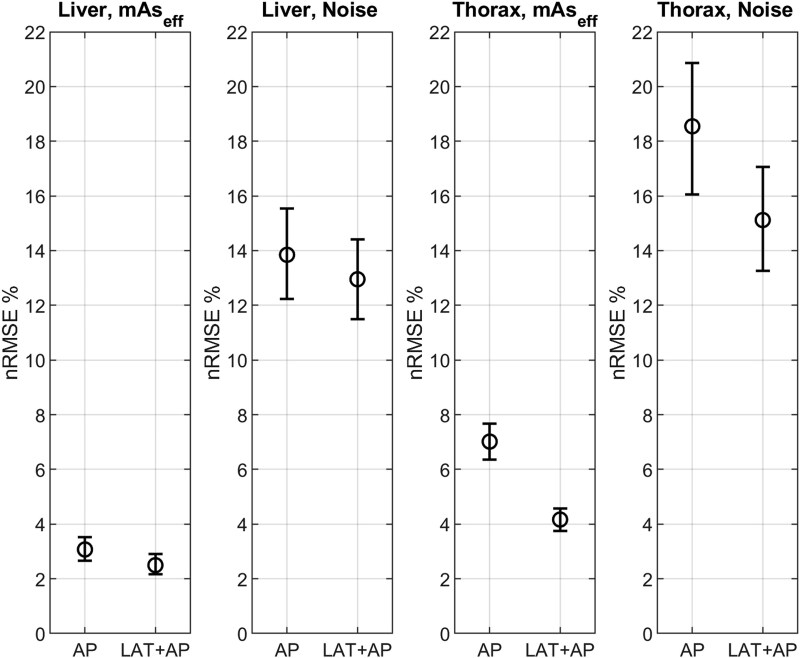
nRMSE with 95% CI for the two groups in the liver and the thorax regions.

Figure 2 presents mAs_eff_ and image noise as a function of D_w_. Figure 3 shows the nRMSE with 95% CI for the two groups in the liver and thorax regions. The LAT + AP group had a statistically significant lower nRMSE for mAs_eff_ and image noise in the thorax region and for mAs_eff_ (but not image noise) in the liver region. The mean difference (with 95% CI) in nRMSE between the AP and LAT + AP groups was: 0.57% (0.012%–1.2%) for liver mAs_eff_, 0.88% (−1.4%–3.0%) for liver noise, 2.9% (2.1%–3.6%) for thorax mAs_eff_, and 3.4% (0.34%–6.5%) for thorax noise.

### Post-hoc finding

**Figure 4:**
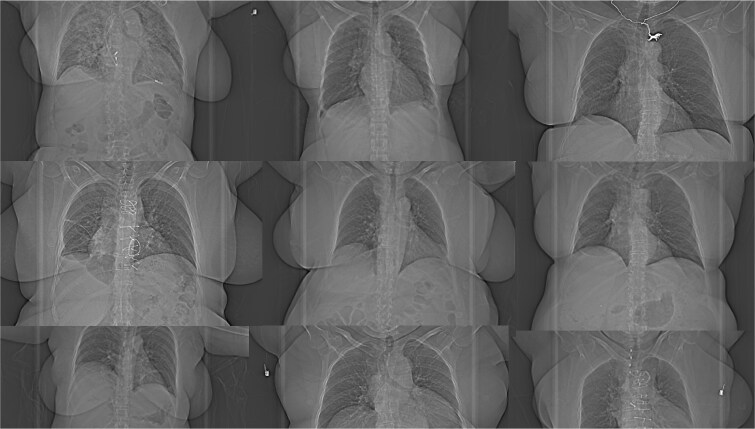
Examples of typical patients from the breast subgroup.

When examining outlier patients in the thorax region (Fig. 2), it was observed that female patients with laterally protruding breast tissue (as seen in the AP localizer) were overrepresented. To investigate this further, a script was developed to randomly (and blindly) display an AP localizer from either group. For each localizer, the reader (DT) determined whether the patient exhibited this anatomical characteristic (Fig. 4).

**Figure 5:**
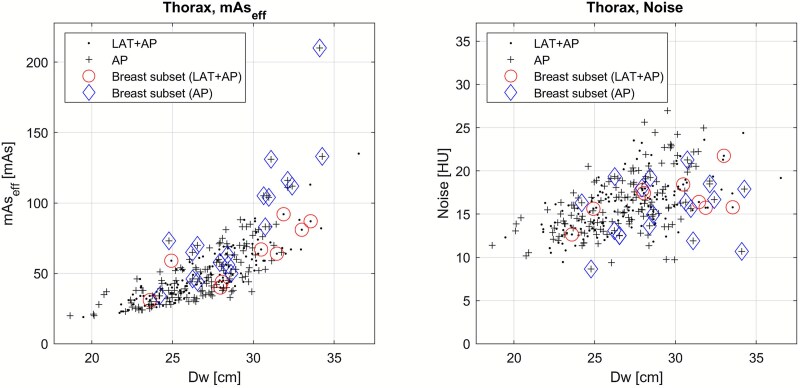
Effective mAs and image noise as a function of D_w_ for both groups in the thorax region (same data as in Fig. 2), with patients from the breast subgroup indicated by circles and diamonds.

Figure 5 presents the results for the thorax region again, with additional data highlighting which patients belonged to the breast subgroup. The graph suggests a particularly large difference in mAs_eff_ for this specific subgroup, with the AP group getting higher mAs_eff_ values. Furthermore, an examination of the image noise graph indicates that these AP patients also exhibited lower noise levels, suggesting that the increased mAs_eff_ was unwarranted. The mean image noise level of this subgroup of the AP group was 2.7 HU lower than expected, measured as the distance from the regression line for the non-breast-subgroup patients (i.e. full data set minus the breast subgroup). This corresponded to an estimated mean radiation dose ~57% higher than needed for all individual cases to achieve the expected image noise level. This deviation from the expected image noise was statistically assessed by incorporating a dummy variable as a second predictor in a linear regression model: image noise ~1 + D_w_ + Dummy, where the dummy variable was set to 1 for patients in the breast subgroup and 0 otherwise. The coefficient for the dummy variable was significant in the AP group (p = 0.0015) but not in the LAT + AP group (p = 0.45). A total of 27 patients (18 AP and 9 LAT + AP) belonged to the breast subgroup, representing ~20% of the female patients in the total dataset.

### Constancy control

All tests were successfully completed within the required tolerance levels.

## Discussion

The results presented for the specific CT model and software version evaluated in this study indicate that ATCM consistency is higher when using LAT + AP localizers compared to AP-only. However, the difference is small, except for a female subgroup in the thorax region with laterally protruding breast tissue. The largest difference between the groups was found in the thorax region, and even when excluding the breast subgroup, the results remained statistically significant for mAs_eff_ but not for image noise. In the thorax region, the range of mAs_eff_ values was larger for the AP group across all patient sizes. For example, within the size range of D_w_ 24–30 cm, the mAs_eff_ range (5^th^–95^th^ percentile) was ~0.9 times the mean mAs_eff_ for the AP group and 0.55 times the mean mAs_eff_ for the LAT + AP group (excluding the breast subgroup). This means that if the average mAs_eff_ is 100, the observed range of mAs_eff_ values would be ~55–145 for AP and 73–128 for LAT + AP. While this difference may be only marginally perceptible in terms of image noise variation, the discrepancies observed in the breast subgroup, if genuine, could be clinically significant. The mean image noise difference alone would likely be noticeable, and the extreme cases could be highly conspicuous.

The main outcome is logical, as LAT + AP localizers provide the software with more data regarding patient transmission and reduce the influence of magnification effects caused by miscentring [11, 12]. The post-hoc finding of a particularly large difference in the thorax region for women with laterally protruding breasts is probably due to the increased anatomical complexity and irregularity in this region. CARE Dose 4D is a proprietary algorithm, and its exact mechanisms are not publicly disclosed. However, it is known to employ more advanced methodologies than simple metrics like LPS measurements to determine appropriate mAs_eff_ [5]. If the algorithm places substantial weight on apparent patient width, the presence of laterally protruding breasts might lead to an overestimation of patient size, consequently increasing radiation dose. However, this remains speculative and given that the finding was post-hoc with a limited number of cases, replication is necessary before drawing firm conclusions.

This study has several limitations. Firstly, D_w_ does not account for patient shape, particularly lateral width, which predominantly influences image noise formation [13, 14]. As a result, identical D_w_ values may yield different mAs_eff_ values depending on variations in patient shape [15]. Although the thorax and liver positions in this study are assumed to have similar average shapes across both groups, the larger range of mAs_eff_ values in the AP group could plausibly also be interpreted as the ATCM being more responsive to changes in shape not captured by the D_w_ measurement. However, this interpretation seems unlikely since all observed patterns remain if mAs_eff_ and image noise are plotted against lateral diameter (measured in the axial image) or LPS (measured in the localizer) (graphs not shown in the manuscript).

Secondly, a protocol with lower pitch (e.g. 0.6) would have been better to assess the consistency of image noise level, since that would have reduced inter-slice variance compared to the current pitch of 1.2. Increased inter-slice variance introduces additional randomness in image noise levels, potentially obscuring differences between the groups. This may explain the wider confidence intervals observed in image noise measurements and why the noise difference was not significant in the liver region.

Thirdly, the lack of a dedicated full-FOV or extended-FOV reconstruction for D_w_ calculations was a limitation. It was not anticipated that so many patients would be cropped due to the manually set FOV. Three potential solutions were considered to address this issue: (1) imputing missing D_w_ values using LPS, (2) calculating D_w_ on cropped images (leading to underestimations of D_w_), or (3) excluding cropped patients entirely (which could introduce bias into the dataset). Given the minimal difference between estimated and measured D_w_, imputation via LPS was deemed the best approach. The highest number of imputations occurred in the thorax region, as technicians prioritized optimal FOV for lung assessment, often disregarding whether peripheral fat was cropped.

There are advantages and disadvantages to using two localizers. The advantages include improved verification of patient centring, more precise scan length prescription, and as suggested by these results increased consistency in image noise and mAs_eff_. The disadvantages include the additional radiation dose from a second localizer and a minimal increase in examination time. However, in the case of thorax examinations, this added dose may be counterbalanced by dose savings in the CT scan. A rough estimate of the increased effective dose by a 57% too high mAs_eff_ in the breast region (which was the mean value for the breast-subset) yields an effective dose increase of ~3 mSv (calculated using the software CT-Expo V 2.4 [16], with a 10 cm region over the breast and a nominal CTDI_vol_ of 10 mGy). Since this applies to only 30 patients, corresponding to 10% of the total cohort (20% of the female patients), the overall average dose increase per examination is ~0.3 mSv, which is comparable to the 0.2 mSv dose from an additional lateral localizer [17]. Thus, it may be advisable to use LAT + AP localizers in thorax region. However, it is crucial to be aware that it may be necessary to adjust the quality reference mAs, and to be aware that these results do not necessarily apply to the latest software versions (VA62/VBxy) available on some newer models. Newer versions of CARE Dose 4D may provide enhanced accuracy of the estimated D_w_ in the thorax region, with the calculation based on a frontal localizer. When a lateral localizer is available, it is used for geometric correction of patient positioning. The benefit is higher consistency between single- and dual-localizer modes.

## Conclusion

For the CT model and software version evaluated in this study, using LAT + AP yielded more consistent mAs_eff_ and image noise, particularly in female patients in the thorax region. The proposed methodology provides a template for evaluating the impact of localizer selection on ATCM consistency. The principal steps are as follows:

1. Select ATCM performance metrics: mAs_eff_ and/or pixel noise.
2. Acquire phantom or patient scans with different localizer orientations, adjusting acquisition settings if necessary to ensure that the mean CTDI_vol_ is equal across the groups.
3. Extract or calculate a size metric, such as water equivalent diameter, for each patient scan.
4. Perform linear regressions of ATCM performance metric against the size metric (transforming the dependent variable if necessary to ensure a linear dependence).
5. Calculate the normalized RMSE for the performance metric using the fit residuals.
6. Test for statistically significant differences in this metric between localizer orientation selections.

This approach would be straightforward to implement in a larger collaboration including all vendors and could potentially inform future guidelines.

## References

[1] Kalender WA, Buchenau S, Deak P. et al. Technical approaches to the optimisation of CT. Phys Med 2008;24:71–9. 10.1016/j.ejmp.2008.01.012. 18331808

[2] Merzan D, Nowik P, Poludniowski G. et al. Evaluating the impact of scan settings on automatic tube current modulation in CT using a novel phantom. Br J Radiol 2017;90:20160308. 10.1259/bjr.20160308. 27845559 PMC5605012

[3] Paolicchi F, Bastiani L, Negri J. et al. Effect of CT localizer radiographs on radiation dose associated with automatic tube current modulation: a multivendor study. Curr Probl Diagn Radiol 2020;49:34–41. 10.1067/j.cpradiol.2018.12.010. 30704769

[4] Söderberg M . Overview, practical tips and potential pitfalls of using automatic exposure control in CT: Siemens CARE dose 4D. Radiat Prot Dosim 2016;169:84–91. 10.1093/rpd/ncv459. 26567324

[5] Rizzo S, Kalra M, Schmidt B. et al. Comparison of angular and combined automatic tube current modulation techniques with constant tube current CT of the abdomen and pelvis. AJR Am J Roentgenol 2006;186:673–9. 10.2214/AJR.04.1513. 16498094

[6] Siemens AG Medical Solutions . Somatom Definition Flash – System Owner Manual. Technical Description. Forchheim: Germany, 2012.

[7] Wang J, Duan X, Christner JA. et al. Attenuation-based estimation of patient size for the purpose of size specific dose estimation in CT. Part I. Development and validation of methods using the CT image. Med Phys 2012;39:6764–71. 10.1118/1.4754303. 23127070

[8] AAPM Task Group 220 . Use of water equivalent diameter for calculating patient size and size-specific dose estimates (SSDE) in CT. American Association of Physicists in Medicine, 2014. Alexandria, VA, USA. PMC4991550 27546949

[9] Wang J, Christner JA, Duan X. et al. Attenuation-based estimation of patient size for the purpose of size specific dose estimation in CT. Part II Implementation on abdomen and thorax phantoms using cross sectional CT images and scanned projection radiograph images Med Phys 2012;39:6772–8. 10.1118/1.4757586. 23127071

[10] International Electrotechnical Commission (IEC) . Evaluation and routine testing in medical imaging departments - part 2-6: Constancy tests ­imaging performance of computed tomography X-ray equipment. IEC 2006;61223-2-6:1–67.

[11] Funama Y, Taguchi K, Awai K. et al. Image noise and radiation dose using an automatic tube current modulation technique at 64-detector computed tomography: effect of off-center patient position, bowtie filter type, and scan projection radiograph. J Comput Assist Tomogr 2009;33:973–7. 10.1097/RCT.0b013e31819d6f6f. 19940670

[12] Kaasalainen T, Palmu K, Reijonen V. et al. Effect of patient centering on patient dose and image noise in chest CT. AJR Am J Roentgenol 2014;203:123–30. 10.2214/AJR.13.12028. 24951205

[13] Kalra MK, Maher MM, Toth TL. et al. Techniques and applications of automatic tube current modulation for CT. Radiology. 2004;233:649–57. 10.1148/radiol.2333031150. 15498896

[14] McCollough CH, Bruesewitz MR, Kofler JM. CT dose reduction and dose management tools: overview of available options. Radiographics. 2006;26:503–12. 10.1148/rg.262055138. 16549613

[15] Burton CS, Szczykutowicz TP. Evaluation of AAPM reports 204 and 220: estimation of effective diameter, water-equivalent diameter, and ellipticity ratios for chest, abdomen, pelvis, and head CT scans. J Appl Clin Med Phys 2018;9:228–38. 10.1002/acm2.12223 PMC5768014 29178549

[16] Stamm GD, Nagel HD. CT-expo - ein neuartiges Programm zur Dosisevaluierung in der CT. Röfo Fortschritte Auf Dem Gebiet Der Röntgenstrahlen Und Der Bildgebenden Verfahren 2002;174:1570–6. 10.1055/s-2002-35937. 12471531

[17] Schmidt B, Saltybaeva N, Kolditz D. et al. Assessment of patient dose from CT localizer radiographs. Med Phys 2013;40:084301. 10.1118/1.4813296. 23927364

